# Supplementary material for: Comparison of long-term patient-reported outcome and quality of life 10 years after severe trauma versus polytrauma
Source: PLoS One. 2026 Mar 25;21(3):e0345816. doi: 10.1371/journal.pone.0345816 (PMC13016348; doi:10.1371/journal.pone.0345816)
Supplement: Attachment — Submitted filename: Response to reviewers_22.01.2026.docx [file pone.0345816.s002.docx]

**Authors’ Response Letter**

| **Reviewer #1’s comment** | **Authors’ response** | **Changes in the manuscript** |
| --- | --- | --- |
| As the authors have correctly mentioned, the low response rate is affecting the quality of the study as well as the accuracy of the inferences made based on the results. with this it is difficult to draw conclusions based on this sample. | The authors would like to thank Reviewer #1 for their feedback and comments provided. The low response rate is caused by the very long follow-up of 10 years and comparable drop-out rates were reported in previous studies with a similar follow-up. | The following was added in the Discussion section:  A further limitation of this study is the low response rate. This is mainly caused by the very long follow-up of 10 years and comparable drop-out rates were reported in previous studies with a similar follow-up. [26, 27] |

| **Reviewer #2’s comment** | **Authors’ response** | **Changes in the manuscript** |
| --- | --- | --- |
| Table 2: The percentage for chest for polytrauma – is shown as "61.2%". Similar to “Face”. It’s likely a copy paste error, please correct it (should be 24.5% for PT based on 12/49) | Thank you very much for pointing this out. We apologize for the error. | The percentage has now been corrected in the revised manuscript. |
| In the demographic data (Table 1), males show a significant percentage among those who were injured compared to females. It is better if this can be discussed meaningfully in the discussion. | We absolutely agree and thank the reviewer for this important comment. The significant predominance of male patients has now been meaningfully discussed in the Discussion section. We contextualize this finding as a well-established epidemiological characteristic of severely injured and polytraumatized populations, supported by trauma registry data and previous studies. | A predominance of male patients was observed in our cohort, particularly among the severely injured group. This finding is consistent with well-established epidemiological patterns in trauma populations, where men are more frequently affected by high-energy trauma mechanisms such as traffic accidents, occupational injuries, and other risk-related activities. Similar sex distributions have been reported in national and international trauma registries, including the German TraumaRegister DGU®, which shows that approximately 70% of severely injured patients are male. The study thus reflects a sex distribution that is typical for severely injured and polytraumatized populations. [28,29] |
| Death – The statement says ‘The survival rate was "50.0% (n=42) SI vs 55.1% (n=49) in the PT group". But these are the responders (42+49=91), not all patients. Calculating survival rate this way leads to major bias. | Thank you for this comment. We agree that the original wording was misleading and could introduce bias. We have therefore revised the text to clarify that the reported survival rates refer only to patients who survived the initial trauma and treatment. | The survival rate of the patients that survived the initial trauma and treatment was 50.0% (n=42) in the severely injured group and 55.1% (n=49) in the polytrauma group. |
| Conclusion – • From the initial sample of 503 participants, after accounting for the 80 deaths we have 423 survivors. However, the responders are only 91 (21%). Drawing recommendations and conclusions from this limited subset likely excludes the worst outcomes, introducing survivorship bias, a limitation that is not acknowledged in the conclusion | We thank the reviewer for this important comment. We agree that drawing conclusions from a limited subset of survivors may introduce survivorship bias. We have therefore revised the Conclusion section to explicitly acknowledge this limitation and to place greater emphasis on the need for further research that can provide more robust and definitive insights into long-term outcomes after severe injury and polytrauma. In addition, the relevance and potential impact of survivorship bias are now explicitly discussed in the Discussion section. | While polytrauma patients exhibit greater physical impairments and functional deficits, psychological and social dimensions appear to be influenced by factors beyond injury severity alone. These findings highlight important associations relevant to long-term outcomes and underscore the need for further research to better define how persistent pain, functional limitations, and psychosocial factors interact in long-term recovery after severe injury and polytrauma. |
| "emphasize the need for individualized rehabilitation" however this study design can't support causal claims |  |  |

| **Reviewer #3’s comment** | **Authors’ response** | **Changes in the manuscript** |
| --- | --- | --- |
| This manuscript addresses an important and under-researched topic by examining long-term patient-reported outcomes and mortality ten years after severe trauma and polytrauma. The extended follow-up period and use of validated outcome measures, together with registry-based ascertainment of mortality, represent notable strengths. | We thank the reviewer for highlighting the strengths of our study. We appreciate the recognition of the extended follow-up period, the use of validated patient-reported outcome measures, and the registry-based mortality data. | - |
| Several methodological and reporting issues, however, require clarification to improve transparency and interpretability. The response rate among surviving patients is low (21.5%), raising substantial concerns regarding selection and non-response bias. The manuscript would benefit from clearer reporting that allows readers to assess whether responders differ systematically from non-responders, as well as a more detailed discussion of the potential direction and magnitude of any resulting bias. A baseline comparison of responders versus non-responders among surviving patients (e.g., age, sex, ISS, ICU stay, mechanical ventilation, major injury regions, if available) should be provided. | We absolutely agree with the statement that a comparison between the responders and non-responders would give important insights regarding the extent to which the findings can be extended beyond the study sample. We acknowledge that the low response rate among surviving patients raises concerns regarding selection and non-response bias. Unfortunately, due to data protection regulations, baseline data for patients who did not consent to participate could not be collected. Nevertheless, we have now explicitly addressed this limitation in the discussion section, emphasizing that differences between responders and non-responders may represent an important factor to consider for the interpretation and transparency of the results. | An important consideration in follow-up studies is the potential influence of non-response. However, due to the lack of consent, baseline data for non-responders could not be collected. Consequently, potential systematic differences between responders and non-responders could not be assessed, which may introduce bias and should be considered when interpreting the long-term outcome findings. |
| The study design is not explicitly stated. Clear identification of the study design is important for transparency and alignment with reporting standards for observational studies. | Thank you very much for this comment. We have now added a clearer description of the study design in the Methods section. | This study was designed as a cross-sectional observational study. [A total of 503 eligible patients were contacted by mail] and were asked to complete a questionnaire at a single time point. |
| Analyses are largely unadjusted despite baseline differences between groups and the observational nature of the data. This limits interpretation of between-group comparisons, and the implications of residual confounding should be more clearly addressed. | We thank the reviewer for this important comment. We totally agree that the residual confounding should be addressed more clearly in the manuscript. We have further emphasized the possibility of residual confounding due to the observational study design. However, most of the baseline differences between groups were intentional and central to the study question. Baseline variables such as ISS, ICU length of stay, and GOS differ between groups because they are causally determined by injury severity and represent downstream consequences rather than confounders. Adjusting for these variables could lead to overadjustment and potentially bias the estimates. | In addition to that given by the observational nature of the study, residual confounding by factors not captured in the dataset cannot be entirely excluded. Therefore, between-group comparisons should be interpreted with caution, and the results should be regarded as exploratory rather than causal. |
| Reporting of participant flow, non-responder characteristics, and missing data handling is incomplete. In particular, the manuscript does not describe how missing questionnaire items or incomplete scales were managed during analysis. | We totally agree that the handling of missing data is a critical aspect, as it can substantially influence statistical analyses and the interpretation of results. | Analyses were conducted using available-case analysis. To avoid introducing bias by inputting missing values patients with missing data for the respective variable were excluded from the corresponding analysis. |
| Further clarification is also needed regarding the derivation of EQ-5D-3L index scores and the rationale for the thresholds used to define reduced and severely reduced quality of life (≤0.8 and ≤0.5). Without this information, interpretation and reproducibility are limited. | We also totally agree with that providing details on the derivation of EQ-5D-3L index scores and the rationale for the thresholds is essential for transparency and reproducibility. We have now added a clear explanation in the relevant sections of the manuscript. | The cutoff values were based on normative population data and previous studies using similar cut-offs in trauma populations. [16,17] |
| Several minor issues should also be addressed, including improving consistency in terminology, correcting typographical and formatting errors, clarifying follow-up contact procedures, and correcting figure numbering to follow standard conventions (the use of “Figure 0” is non-standard). | We thank the reviewer for these suggestions and apologize for the errors. We have corrected the errors. | Several minor changes have been incorporated into the manuscript. |
| I have no concerns regarding research ethics, consent procedures, or potential dual publication based on the information provided. Addressing the above issues would substantially improve the clarity and robustness of the manuscript. | We greatly appreciate the reviewer’s constructive feedback, which has been carefully incorporated to strengthen the manuscript. | - |
